# Modification in ICU design may influence circadian serum cholinesterase activities: a proof-of-concept pilot study

**DOI:** 10.1186/s40635-024-00709-5

**Published:** 2024-12-23

**Authors:** Sebastian Schmidt, Maria Heinrich, Klaus-Dieter Wernecke, Claudia Spies, Laura Hancke, Anika Mueller, Alawi Luetz

**Affiliations:** 1 Department of Anesthesiology and Intensive Care Medicine, Charité - Universitätsmedizin Berlin, Corporate Member of Freie Universität Berlin and Humboldt Universität zu Berlin and Berlin Institute of Health, Berlin, Germany; 2 Institute of Biometry and Clinical Epidemiology, Charité - Universitätsmedizin Berlin, Corporate Member of Freie Universität Berlin and Humboldt Universität zu Berlin and Berlin Institute of Health, Berlin, Germany; 3 Department of Healthcare Management, Technische Universität Berlin, Berlin, Germany

**Keywords:** Architecture, Critical care, Nonpharmacological, Circadian rhythm, Cholinesterase, Delirium

## Abstract

**Background:**

Deficits in cholinergic function are assumed to cause cognitive decline. Studies have demonstrated that changes in serum cholinesterase activities are associated with a higher incidence of delirium in critically ill patients. Additionally, basic research indicates that the cholinergic and circadian systems are interconnected, with each system influencing the functionality of the other. This data analysis of a proof-of-concept pilot study investigates whether modification in ICU design, including dynamic light therapy, may influence the circadian oscillation of serum cholinesterase activities.

**Methods:**

We enrolled adult critically ill patients who were on mechanical ventilation and had an anticipated ICU stay of at least 48 h. The patients were treated in either modified or standard ICU rooms. The modified rooms received extensive architectural modifications, including a new dynamic lighting system. Serum acetylcholinesterase and butyrylcholinesterase activities were measured every four hours for up to three 24-h assessment periods.

**Results:**

We included 64 patients in the data analysis (*n* = 34 patients in modified rooms,* n* = 30 in standard rooms). The median values of serum acetylcholinesterase and butyrylcholinesterase activities showed different patterns. Acetylcholinesterase activities differed significantly between the groups during the first assessment period (*p* = 0.04) and the second assessment period (*p* = 0.045). The intensity of light, as quantified by the effective circadian irradiance, significantly influenced the activities of acetylcholinesterase and butyrylcholinesterase throughout all assessment periods for patients in both groups (*p* < 0.001). The analysis showed significant interaction (*p* < 0.001), indicating that the differences in acetylcholinesterase and butyrylcholinesterase activities between the groups were inconsistent over time but apparent during specific periods of the day.

**Conclusion:**

Implementing a comprehensive set of changes to the design of ICU rooms, including a dynamic lighting system, may influence the course of the activity patterns of acetylcholinesterase and butyrylcholinesterase in critically ill patients. Modifications to environmental factors could potentially offer neuroprotective benefits and facilitate the realignment of circadian rhythms within the cholinergic system.

*Clinical trial registration* ClinicalTrials.gov: NCT02143661. Registered May 21, 2014.

## Background

The cholinergic system is a network of neurons that orchestrates cognitive processes, regulates sleep patterns, and influences inflammatory responses [1, 2]. In critically ill patients, the cholinergic system is recognized primarily for its role in the pathophysiology of delirium [3]. A prevalent hypothesis suggests that delirium is often associated with a central cholinergic deficiency resulting from a shortage of acetylcholine (ACh) [4, 5].

In clinical trials, the evaluation of the cholinergic system is performed by measuring serum cholinesterase (ChE) activities [6–8]. This enzymatic activity reflects the dynamics of ACh breakdown, with acetylcholinesterase (AChE) localized to neuronal synapses and red blood cell membranes, and butyrylcholinesterase (BChE) found primarily in plasma. [9].

Recently, ChEs have been the focus of research due to their potential role as biomarkers in perioperative settings, as well as in the monitoring of critically ill patients. The results are not consistent. Increased [7, 10] and decreased [11–13] levels of AChE activity have been associated with an increased risk of postoperative delirium (POD) and delirium during critical illness, while decreased levels of BChE have been associated with increased inflammatory processes [14] and also an increased risk of POD [7, 11, 13]. Another study has revealed that reduced BChE activities measured on hospital admission in trauma patients are correlated with morbidity and a prolonged ICU stay [15].

In these trials, the evaluation of ChE activities has been confined to once a day measurements. This approach has limitations, as it overlooks the circadian rhythm of cholinergic markers. Animal studies have shown that ChE activities fluctuate daily, with high activity during the inactive phase and low levels during the active phase [1, 16].

Basic research highlights that the rhythmicity observed in the cholinergic system is not merely a passive reflection of physiological states but actively responds to environmental signals and behavioral patterns [17, 18]. In this regard, studies in animal models have shown a dynamic interaction between the cholinergic system and the master circadian clock within the suprachiasmatic nucleus (SCN) [1, 19]. This interplay appears to play an important role in orchestrating the regulation of sleep arousal cycles and in maintaining cognitive processes.

Our research group has developed an innovative concept for ICU rooms that incorporates a dynamic lighting system (DLS) [20]. The objective was to create a therapeutic environment that minimizes stressors and aligns with natural circadian rhythms, thus reducing the need for prolonged sedation and allowing patients to benefit from earlier cognitive stimulation and mobilization.

Upon completion of the renovation of two ICU rooms, we conducted a pilot proof-of-concept study. The findings indicated that the redesigned ICU rooms could potentially reduce both the incidence and severity of delirium in patients treated in modified rooms compared to those in standard rooms [21]. Furthermore, the study observed distinct circadian melatonin patterns between patients in the two groups.

Based on these findings, the following analysis focuses on determining whether a redesign in the ICU environment, including dynamic light therapy, changes the levels of AChE and BChE activities compared to standard care and what the effect size of this change is.

## Methods

This analysis uses data from the prospective observational cohort proof-of-concept pilot study ’Evaluation of the New All-New Environment for Critically Ill Patients’ (VITALITY) [21], which was carried out at the Charité - Universitätsmedizin Berlin hospital. The study was registered on ClinicalTrials.gov (NCT02143661. Registered May 21, 2014) and was approved by the Charité Ethics Committee (number EA1/019/14; approval date January 30, 2014 and the institutional data protection officer. Written informed consent was obtained from all patients or their authorized representatives after ICU admission; if consent was initially obtained from a surrogate, we obtained consent from the patient once it was deemed legal competent. All procedures were followed according to the ethical standards of the Humane Ethics Committee of Charité and the Declaration of Helsinki of 1975.

### Study population

The study included patients in the 8i ICU at Campus Virchow Klinikum who were 18 years or older, required mechanical ventilation and had a planned ICU stay (LOS) of at least 48 h. Exclusion criteria were as follows: substantial recent ICU exposure [22], ICU readmission during the current hospital stay, psychiatric or sleep disorders, delirium at admission, analphabetism, amaurosis, anacusis or severe hypoacusis, history of stroke and known residual cognitive deficits, history of cardiopulmonary arrest or pulseless electric activity with cardiopulmonary resuscitation followed by therapeutic hypothermia during the current hospital stay, history or suspicion of hypoxic brain damage, intracranial bleeding (ICB) or elevated intracranial pressure (ICP) 7 days before study inclusion, open chest after cardiac surgery, patients who were unlikely to survive for 24 h, liver-cirrhosis, non-German speaking patients, informed consent could not be obtained or was refused, patients with participation in other clinical trials and patients with accommodation in an institution due to an official or judicial order.

### Interventions

Two rooms of ICU-8i at Campus Virchow-Klinikum received a comprehensive renovation in the architectural room and interior design [20]. Modifications aimed at reducing noise, stress relief, cognitive training, early mobilization, and workflow optimization [23]. In addition, we developed a new DLS for patient-individualized lighting therapy [20, 24, 25]. The terms “standard room” and “modified room” refer to patient rooms that are not altered or equipped with specific modifications, respectively.

Each bed in the modified rooms was equipped with the newly developed DLS. DLS stands for a feature set that enables the control and timing of both the illuminance (EV, lux [lx]) and the correlated color temperature (CCT, kelvin [K]).

The modified rooms achieved a maximum effective irradiance ($E_C$) of 2.5 $W x m^-2$ between 12 pm and 2 pm. Standard rooms were equipped with white-light fluorescent tube ceiling lamps (FT-CL). In standard rooms, patients were exposed to a static $E_C$ of 0.3263 $W x m^-2$. For a detailed description of the light intervention and study procedures, see the primary publication [21].

### Assessment of parameters

Study assessments started the morning after participant enrollment. The clinical examination included a prospective evaluation of the depth of sedation, delirium, and pain, conducted at least every 8 h. The study protocol encompassed three assessment periods (AP-A, AP-B, and AP-C). During these periods, AChE and BChE activities were measured using point-of-care testing (*ChE check mobile*, Securetec Detektions-Systeme AG, Neubiberg, Germany) as previously described [7]. If a patient was ready for discharge from the ICU after completing AP-A or AP-B, no additional assessments were performed.

**Fig. 1:**
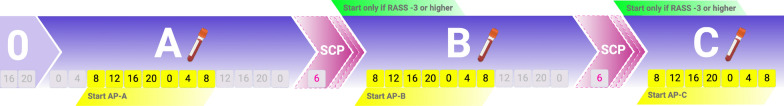
Study flow diagram. 0, day of patient enrollment; AP, assessment period (**A**, 1st day of intervention;** B**, 3rd day of intervention or later;** C**, 5th day of intervention or later.); SCP, sedation check period; RASS, Richmond Agitation-Sedation Scale. Numbers in yellow squares indicate the time of blood collection for serum AChE and BChE activities (e.g., 8 = 08:00 in the morning). Each patient completed AP-A. If a patient was prepared for ICU discharge upon completing either AP-A or AP-B, no further assessments were conducted. Every morning at 6:00 AM, the patient’s depth of sedation (SCP) was evaluated. AP-B and AP-C were only initiated if the patient had a RASS score of -3 or higher. Depending on the patient’s sedation status, multiple days (SCPs) could pass before starting either AP-B or AP-C

Whole blood samples were collected from the arterial line every 4 h, starting at 8:00 AM and continuing until 8:00 AM the following day (resulting in seven study visits per examination period). AP-A started at 8:00 am after inclusion in the study. During the following days of the study, each morning at 06:00 am, the patient’s depth of sedation was evaluated (Sedation Checkup Period [SCP]). AP-B and AP-C began at 8:00 AM only if the patient had a Richmond Agitation-Sedation Scale (RASS) of -3 or higher. Consequently, in some patients, ChE measurements were not performed on consecutive days (Fig. 1).

### Statistical analysis

This is a secondary endpoint analysis of the VITALITY study [21]. Patient characteristics were expressed as median with the 25th percentile and the 75th percentile, or frequencies with percentages. Because of limited sample sizes and/or nonsymmetrically distributed observations, we applied preferably nonparametric statistics. Differences between groups (modified versus standard room) were tested univariately using the exact Mann–Whitney or Fisher test. The median values of the AChE and BChE activities were calculated and represented graphically. The differences in AChE and BChE activities between modified and standard rooms with respect to the entire time course were analyzed using a multivariate nonparametric covariance analysis of longitudinal data in a two-factor design (MANCOVA) (1st factor [independent], groups (rooms); second factor [dependent], study visits; added a metric covariate [$E_C$]) [26]. This results in three tests: differences between groups, significant changes in time, and interactions between groups and time. In total, MANCOVA was applied six times, respectively, for the AChE and BChE examinations in three assessment periods. The MANCOVA culminates in the calculation of relative effect sizes [21]. The relative effect size represents the impact of the categorical factor X (rooms) on the responses Z (AChE, BChE) under the influence of the covariate $E_C$ (on a scale between − 1 and 1), therefore the intrinsic effect of the treatment (rooms with different lighting conditions). Finally, in order to examine the results of the multivariate nonparametric procedures, generalized estimating equations [GEE] were applied as a parametric pendant [27]. The *p*-values achieved are to be understood as exploratory in the sense of the pilot study, i.e., do not allow any confirmatory generalization. Moreover, all tests should be understood as constituting exploratory data analysis, in that no adjustments for multiple testing were made.

## Results

**Fig. 2:**
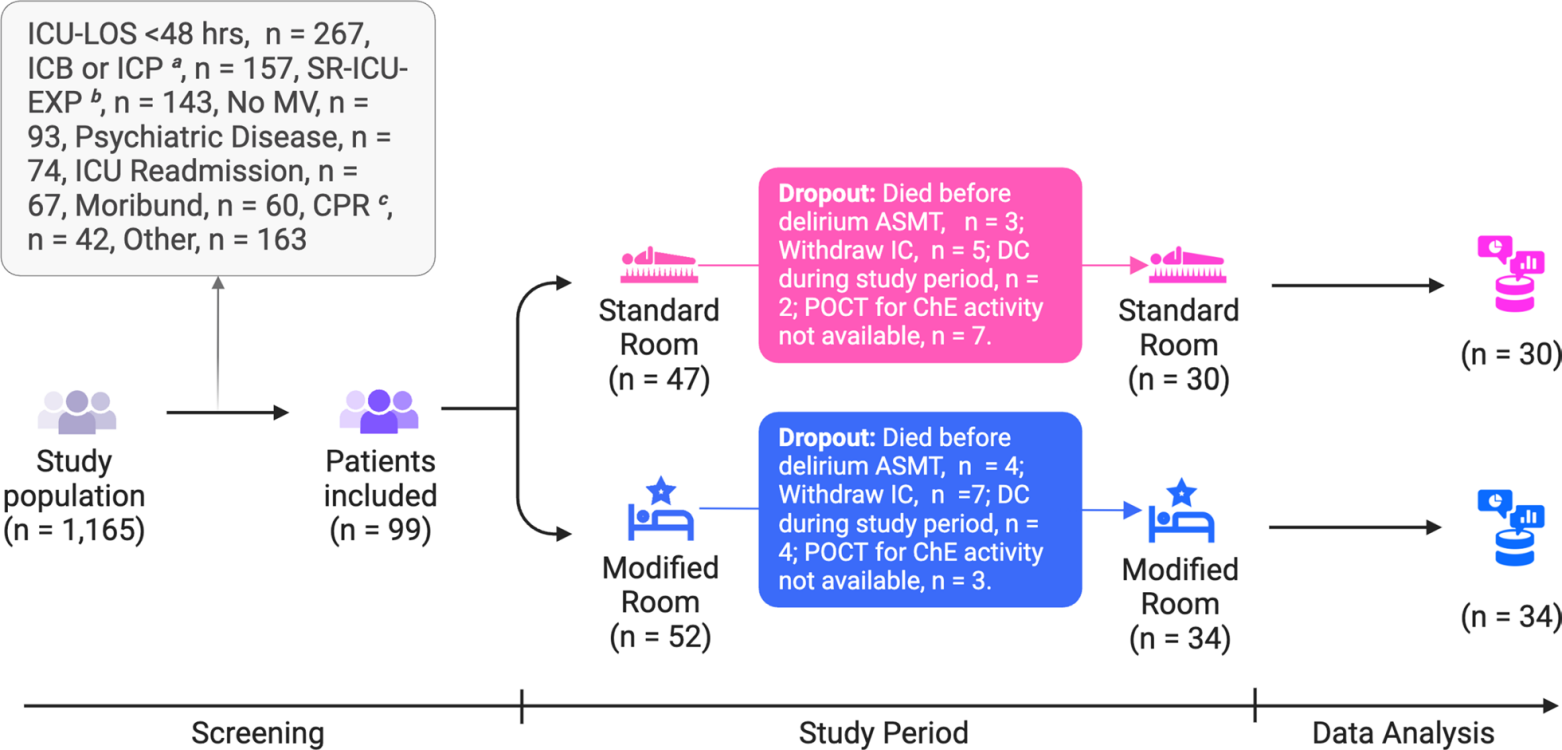
Patient enrollment screening and eligibility flow diagram.* ICU-LOS* intensive care unit length of stay,* ICB* intracranial bleeding,* ICP* intracranial pressure,* SR-ICU-EXP* substantial recent intensive care unit exposure,* MV* mechanical ventilation,* CPR* cardiopulmonary resuscitation,* ASMT* assessment,* IC* informed consent,* DC* discharge, ^a^7 days before study inclusion; ^b^Patients with substantial recent ICU exposure: i.e., receipt of mechanical ventilation in the 2 months before the current ICU admission, $>5$ ICU days in the month before the current ICU admission, or $>72$ h with organ dysfunction during the current ICU admission; ^c^Patients with a history of cardiopulmonary arrest or pulseless electric activity with cardiopulmonary resuscitation followed by therapeutic hypothermia during current hospital stay; POCT, point-of-care testing; ChE, cholinesterase

We screened 1165 patients between June 28, 2014, and June 1, 2017. A total of 99 patients were enrolled in the VITALITY study. The most common reasons for screening failure were an anticipated stay in the ICU of less than 48 h (*n* = 267), the presence of an intracranial bleed or elevated intracranial pressure (*n* = 157), and significant recent exposure to the ICU (*n* = 143). In total, 12 patients or their representatives withdrew informed consent during study evaluations. Three patients in the standard rooms and four in the modified rooms died before the first valid delirium evaluation could be obtained. In addition, six patients were discharged before the study period was completed due to internal capacity allocation decisions made by the lead ICU physician. Intermittent maintenance work on the point-of-care device prevented the initiation of ChE value measurements in 10 patients. Thus, ChE activity values were analyzed for a total of 64 patients in AP-A. Due to the requirement of sedation status at the beginning of the second and third assessment periods and the dependence on the duration of treatment in the ICU, 44 patients could still be analyzed with respect to ChE activity in AP-B and 38 patients in AP-C (Fig. 2). There were no differences in patient characteristics or outcome parameters between patients treated in the standard room compared to the modified room (Table 1).

**Table 1:** Patients characteristics and outcome parameters

	Modified room (n=34)	Standard room (n=30)	P
Age, *yr*	54.5 (38-66)$^{1}$	57 (41–72)$^{1}$	0.6279$^{2}$
Female,* n*	21	19	1.000$^{3}$
Emergency admission, *n*	23	22	0.785$^{3}$
APACHE II on admission	21 (11–27)$^{1}$	22 (12–28)$^{1}$	0.7105$^{2}$
SAPS II on admission	36.5 (23–51)$^{1}$	39 (30–60)$^{1}$	0.3201$^{2}$
SOFA on admission	7 (5–11)$^{1}$	9 (6–11)$^{1}$	0.3252$^{2}$
Surgical intervention,* n*	20 (58.8)	17 (56.7)	1.000$^{3}$
ICU length of stay, *d*	12.5 (5–24)$^{1}$	13 (9–23)$^{1}$	0.5402$^{2}$
Mechanical ventilation, *h*	216 (46–460)$^{1}$	239.5 (68-508)$^{1}$	0.7358$^{2}$
Hospital length of stay, *d*	27 (18-45)$^{1}$	22.5 (13-40)$^{1}$	0.4719$^{2}$
Discharge to home, *n*	19	13	0.453$^{3}$
In hospital mortality, *n*	2	3	0.673$^{3}$

APACHE II, Acute Physiology and Chronic Health Evaluation II, SAPS II, Simplified Acute Physiology Score; SOFA, Sepsis-related Organ Failure Assessment Score. A $p < 0.05$ was considered significant. $^{1}$Values are presented as medians with an interquartile range (25th to 75th in parentheses). $^{2}$Intergroup analysis: exact Mann–Whitney U test. $^{3}$ Intergroup analysis: Fisher exact test

**Fig. 3:**
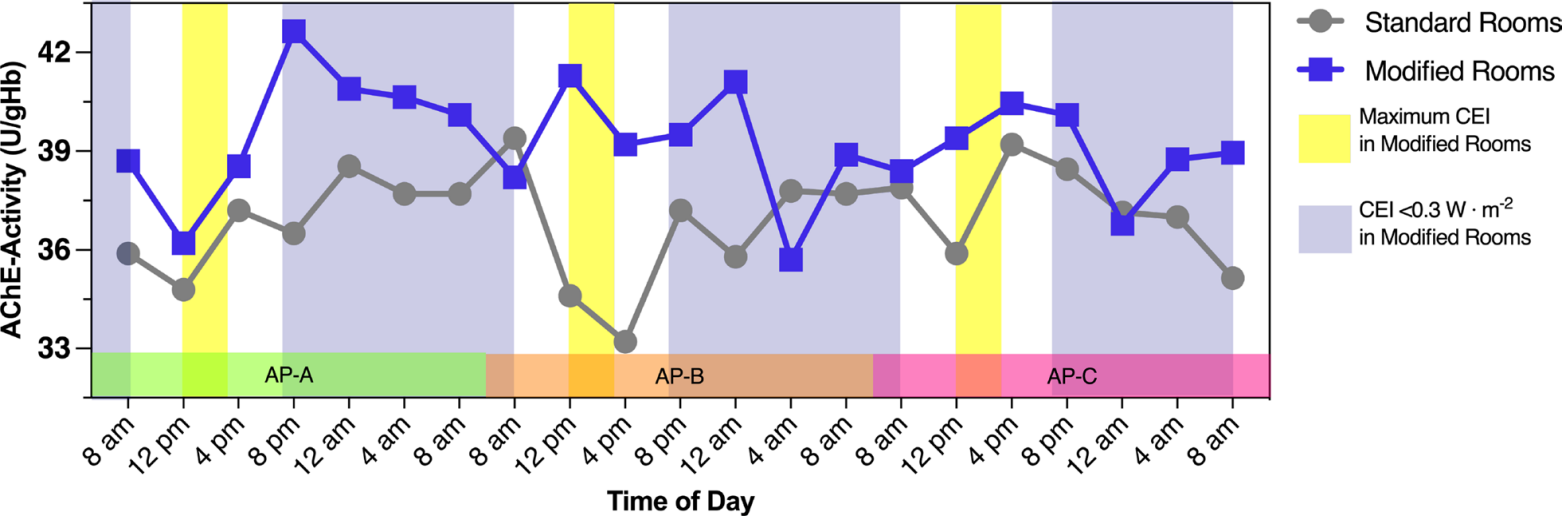
Serum acetylcholinesterase activities presented as medians during assessment periods (AP)** A**,** B** and** C**.* CEI* circadian effective irradiance

The median values of AChE activity showed different circadian patterns depending on the type of room in which the patients were treated, with higher values observed in the patients treated in modified rooms (Fig. 3). Examining the results by GEE resulted in significant group differences in AP-A (*p* = 0.04) and AP-B (*p* = 0.045) for AChE and confirmed other findings.

**Fig. 4:**
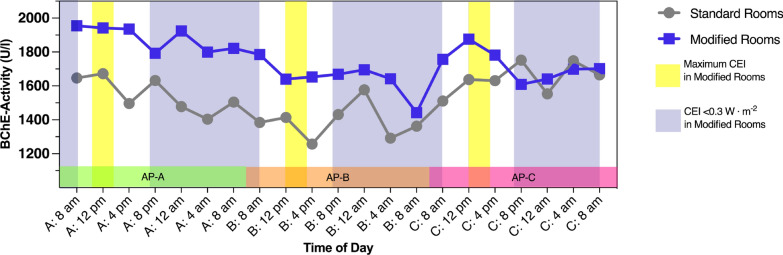
Serum butyrylcholinesterase activities presented as medians during assessment periods (AP)** A**,** B** and** C**.* CEI* circadian effective irradiance

In contrast, the median values of BChE activity showed a minimal circadian fluctuation (Fig. 4). Throughout the progression of AP-A, BChE activities demonstrated a gradual decline. In particular, patients treated in modified rooms consistently maintained higher BChE activities compared to those in standard rooms, but without significant group differences.

**Table 2:** Multivariate test statistics of acetylcholinesterase activities under the influence of circadian effective irradiance

Hypothesis	*p*-values		
	AP-A	AP-B	AP-C
Differences between groups during entire AP	$0.44$	0.79	0.62
Systematic time effect	$< 0.001$	$< 0.001$	$< 0.001$
Interactions: differences do change during AP between groups	$< 0.001$	$< 0.001$	$< 0.04$

AP = assessment period, AP-A = first day of intervention, AP-B = third day of intervention or later, AP-C = fifth day of intervention or later

### Dynamic light intervention and cholinesterase activities

Multivariate nonparametric covariance analysis of longitudinal data revealed that $E_C$ had a significant influence on the course of AChE and BChE activity for AP-A, AP-B, and AP-C ($p < 0.001$) in standard and modified rooms.

The analysis showed no difference in AChE activity between patients in standard and modified rooms during the whole time course of AP-A ($p < 0.44$), AP-B (*p* = 0.79) and AP-C (*p*= 0.62) (Table 2), as well as no difference in BChE activity during AP-A (*p* = 0.48), AP-B (*p* = 0.72) and AP-C (*p*= 0.51) (Table 3).

Patients in both modified and standard rooms showed systematic time effects in AP-A, AP-B, and AP-C, referring to significant changes in AChE and BChE activities over time ($p < 0.001$) (Tables 2, 3).

Furthermore, there were significant interactions in AChE ($p< 0.001, p < 0.001, p = 0.04$) and BChE ($p< 0.001, p< 0.001, p < 0.001$) activities for all APs, revealing that differences between rooms are not the same over time, but particularly pronounced at specific time periods (Table 2, 3).

**Fig. 5:**
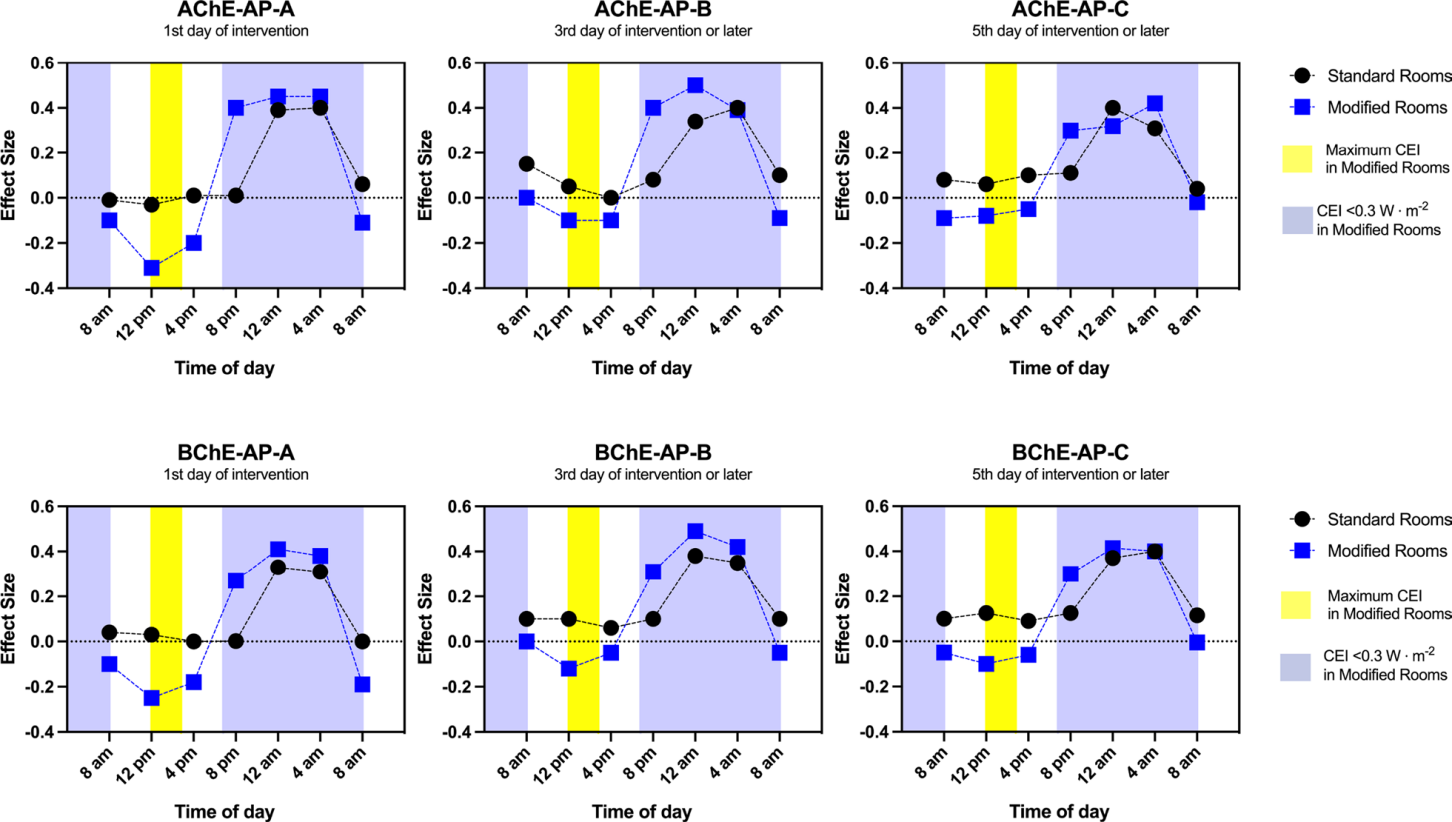
Relative effect sizes of serum acetylcholinesterase and butyrylcholinesterase activities under the influence of circadian effective irradiance (Ec ). AP, assessment period: AChE-AP-A and BChE-AP-A, 1st day of intervention; AChE-AP-B and BChE-B, 3rd day of intervention or later; AChE-AP-C and BChE-AP-C, 5th day of intervention or later

For a graphical representation of the MANCOVA results, the calculated values of the relative effect sizes are plotted in Fig. 5 for both groups and each time point. Differences in the relative effect sizes between rooms are distinctly observable from 08:00 am to 04:00 pm during AP-A, but these differences between groups are getting smaller during AP-B and AP-C.

**Table 3:** Multivariate test statistics of butyrylcholinesterase activities under the influence of circadian effective irradiance

Hypothesis	*p*-values		
	AP-A	AP-B	AP-C
Differences between groups during entire AP	0.48	0.72	0.51
Systematic time effect	$< 0.001$	$< 0.001$	$< 0.001$
Interactions: differences do change during AP between groups	$< 0.001$	$< 0.001$	$< 0.001$

AP = assessment period, AP-A = first day of intervention, AP-B = third day of intervention or later, AP-C = fifth day of intervention or later

## Discussion

We found significant group differences in AChE activity during assessment periods A and B, with generally higher values observed in patients treated in modified rooms. AChE activities showed more pronounced fluctuations during 24-h periods, in contrast to BChE activities. BChE activities did not show significant differences between the groups. However, median values tended to be higher in patients treated in modified rooms. Regarding the influence of light, the results of our study demonstrated that $E_C$ significantly impacted the activities of AChE and BChE in both groups during all evaluation periods. Furthermore, the analysis revealed significant interactions, indicating that differences in the activities of AChE and BChE between the groups fluctuated over time, but became particularly noticeable at specific periods of the day.

Previous research on AChE and BChE activities has mainly focused on perioperative settings, examining their potential as biomarkers of delirium [7, 11, 12]. Although these studies suggest a link between ChE activities and POD, precise mechanisms are not yet fully understood. Adam et al. [11] found that patients who developed POD after cardiac surgery had significantly lower preoperative AChE activity compared to those who did not experience POD. This observation supports the prevailing hypothesis that reduced AChE activity is associated with the onset of POD. It is proposed that a deficiency in AChE activity impairs the enzyme’s ability to effectively break down the neurotransmitter ACh in the synaptic cleft. As a result, the termination of stimulus transmission is hindered, preventing the initiation of new stimulus transmissions [11]. Interestingly, our results revealed significantly higher AChE activity in patients treated in modified rooms. This finding, in line with Adam’s research, suggests that room modifications may have potential positive effects on the cholinergic system. Adam et al. also found that a decrease in BChE activity on postoperative day one was associated with the occurrence of POD. Furthermore, BChE activity decreased significantly throughout the perioperative period in the overall study population. Referring to a study by Zivkovic et al. [14], who also observed a reduced BChE activity after surgery, they suggested that cholinergic modulation of the inflammatory response might be responsible for this effect. In line with this hypothesis, our results indicate a decrease in the median values of BChE activity during the early treatment phase, with a tendency towards lower values in patients treated in standard rooms. In the later treatment phase during AP-C, BChE activities showed an upward trend, possibly due to the resolution of the acute phase of the disease.

In line with these observations, a recent study involving 272 patients found that a reduction in BChE activity in the early stages of critical illness was independently linked to a greater severity of acute brain dysfunction. This was indicated by fewer days of survival without delirium or coma [10]. In contrast to Adam et al., this study revealed that higher AChE activity on a given day was associated with higher odds of being delirious. However, it should be noted that this association was not observed when AChE activity was normalized to hemoglobin levels, a common practice in previous research and one that was also employed in our study. The authors concluded that altered ChE activities might indicate underlying mechanisms of acute brain organ dysfunction.

In line with this conclusion, Zujalovic et al. [28] discovered in a study involving 175 critically ill patients that approximately 90 % of septic patients with suspected septic-associated encephalopathy exhibited a statistically significant time-dependent fluctuation—increase or decrease—in AChE activity over a period of at least five consecutive days. In particular, most of these patients demonstrated a decrease in AChE activity. In contrast, no change in AChE activity was observed in nonseptic patients, even those experiencing delirium.

One major limitation of the studies conducted so far is the restriction to measurements taken once a day that do not capture the circadian course of cholinergic activity. Experimental animal studies provide evidence that AChE activities exhibit circadian periodicity in concert with other cholinergic markers [1, 16]. In the clinical context, the interaction between the cholinergic and circadian systems has been explored mainly within dementia-related research [29]. Studies on Alzheimer’s disease (AD) have shown that circadian rhythmicity in the cholinergic system is crucial for optimal memory processing [30]. The loss of this rhythmicity is believed to contribute to cognitive impairments. Support for this pathophysiological mechanism has been demonstrated through a study involving human participants, which revealed that the physiological decrease in ACh levels during slow-wave sleep plays a crucial role in the consolidation of declarative memory [31].

Beyond its role in sleep, the cholinergic system also appears to play a significant role in the regulation of circadian processes [17]. Data from basic research indicate that the circadian system can be actively entrained by external factors and behaviors through cholinergic signaling. This process may involve two mechanisms: light exposure and cognitive activities. Light has been shown to potentially increase the concentration of ACh in the SCN, contributing to the adjustment of circadian rhythm [32]. Cognitive activities have been identified as powerful non-photic zeitgebers, which can influence circadian rhythms through cholinergic modulation of the central pacemaker [19]. These findings underscore the complex interplay between environmental signals, cognitive stimulation, and cholinergic signaling in the regulation of the body’s internal clock. This interaction is complex and not fully established, but may be particularly significant in critically ill patients prone to chronodisruption and delirium [33–36].

The focus of managing these conditions has changed to non-pharmacologic approaches, including modifications to interior and architectural spaces [37]. In this context, the publication of our analysis’s primary study revealed a higher risk of developing delirium and a higher severity of delirium in patients in standard rooms, compared to those in modified rooms [21]. In addition, the study results revealed different circadian melatonin patterns between patients in both groups. The authors concluded that dynamic light therapy may potentially influence delirium outcomes by modulating circadian melatonin levels.

One possible key point in interpreting these results in relation to our analysis is the finding of basic research that melatonin, along with its derivatives and metabolites, may modify AChE activities [38, 39]. Therefore, differences in melatonin levels between patients treated in modified and standard rooms could explain the differing AChE activities observed between the groups. This finding indicates that optimizing melatonin levels through environmental modifications could be a potential strategy to enhance cholinergic function in critically ill patients. The significance of clinical outcomes, such as delirium, along with potential preventive and therapeutic strategies, should be addressed in further studies.

Another factor contributing to the observed differences in AChE and BChE activities between the groups could be the varying photometric parameters of DLS and FT-CL [20, 24]. Therefore, we continue to analyze the activities of AChE and BChE under the influence of the different $E_C$. The results of our study demonstrated that $E_C$ significantly impacted the activities of AChE and BChE in patients treated in modified and standard rooms in AP-A, AP-B, and AP-C. In addition, the results of our study revealed that the differences in relative effect sizes between rooms varied over time, with particularly pronounced variations during specific time periods. The differences were most noticeable between 8:00 AM and 4:00 PM. During this period, variations in lighting conditions between different types of rooms were especially pronounced.

Building on our primary publication, these findings contribute to a growing body of evidence on the key factors that lead to improved patient outcomes in modified ICU rooms. A more comprehensive understanding of these elements can support the foundation of concepts for evidence-based ICU room designs. Such designs may employ various solutions depending on the unique needs of specific ICU cohorts, the cultural diversity between nations, and the distinct attributes of healthcare systems, including the unique obstacles faced by low-income countries.

### Strength and limitations

The main strength of our study is that we evaluated the course of ChE activities for patients between rooms in a two-armed parallel group design for three AP. Furthermore, we applied MANCOVA, a statistical approach that meets the needs of time course and interaction. We decided to focus on the impact of $E_C$, as the light intervention is considered a critical factor in restoring and maintaining circadian rhythms in our study participants. However, some important limitations must be considered. A major limitation was the open-label design without randomization. However, the groups are homogeneous, so we assumed that no adjustment was necessary for further confounders. Considering that our study evaluates the effects of a multi-component design intervention, the observed changes in AChE and BChE activity may not only be attributed to different levels of $E_C$ in rooms. The DLS offers features that expand beyond bright light therapy, including visual aids for reorientation and modules for active cognitive training [20, 25]. Furthermore, peripheral ChE activities may not correlate with central ChE activities. In this study, measurement of central ChE activities, e.g., in cerebrospinal fluid, was not possible due to its invasiveness. Thus, the values of peripheral ChE can only be considered as an approximation. Finally, missing data on anticholinergic burden is a limitation. However, recent studies highlighted the absence of a validated scale to accurately quantify cumulative anticholinergic effects [40, 41]. These studies also did not find a significant link between preoperative anticholinergic load and postoperative delirium, nor any correlation between anticholinergic load and ChE activity.

## Conclusions

Data from this proof-of-concept pilot study revealed that the median AChE activities fluctuate during the diurnal cycle in critically ill patients, while the median BChE activities exhibit only minor fluctuations. The graphical representation of calculated relative effect sizes underscores that the variations in ChE activities did not remain constant throughout a 24-h cycle. Instead, they were significantly pronounced when the light intensity in the modified rooms peaked. This suggests that light therapy as part of a multi-component room concept contributed to the observed differences in ChE activities between groups, which may provide neuroprotective benefits. More studies are needed to assess the significance of altered ChE rhythms in critical illnesses. These findings could improve therapeutic strategies, including chronobiologically oriented interventions.

## Data Availability

All data generated or analyzed during this study are included in this published article and its Additional file. The datasets used and/or analyzed during the current study are available from the corresponding author on reasonable request.
